# A proposal for coherent nomenclature of multicomponent crystals

**DOI:** 10.1107/S2052520618015858

**Published:** 2019-01-23

**Authors:** Marlena Gryl, Marcin Kozieł, Katarzyna M. Stadnicka

**Affiliations:** a Faculty of Chemistry, Jagiellonian University, Gronostajowa 2, Kraków, 30-387, Poland

**Keywords:** nomenclature, multicomponent materials, cytosine

## Abstract

Here a new, systematic, unambiguous and unified nomenclature for multicomponent materials is presented. The approach simplifies naming schemes of extraordinary co-crystals containing multiple building blocks with different charges. Although the presented examples of cytosine compounds cannot cover all possibilities, they clearly show that the new nomenclature is flexible and can be easily extended to other multicomponent materials.

## Abstract 2

A new, systematic, unambiguous and unified nomenclature applicable for co-crystals and other multicomponent solid materials is presented.

## 1. Introduction   

Multicomponent molecular solids have drawn much attention over the last decade due to the possibility of tuning the properties of materials through cocrystallization. Pharmaceutical co-crystals (Steed, 2013 ▸; Smith *et al.*, 2013 ▸), optoelectronic materials (Gryl *et al.*, 2013 ▸, 2014 ▸; Gryl, 2015 ▸), storage and energetic systems (Bolton & Matzger, 2011 ▸; Aakeröy *et al.*, 2015 ▸; Kent *et al.*, 2018 ▸) are just a few examples of current applications of those materials.

Early approaches toward classification of multicomponent materials can be found in the works of Kitaigorodsky (1973 ▸, 1984 ▸) and Zorkii (1964 ▸; Zorkii *et al.*, 1977 ▸). They introduced the terms ‘mixed crystals’, ‘packing complexes’ and ‘heteromolecular crystals’ to the scientific literature. A more detailed analysis of molecular systems was given by Herbstein (2005 ▸), where materials were classified based on interactions between crystal building blocks. When there was a prevalence of interactions between different components (*A*⋯*B*), materials were considered molecular compounds. All other cases were classified as molecular complexes and included inclusion, segregated stack and packing complexes.

Nowadays multicomponent materials include multiple types of materials including salts, co-crystals, solvates and coordination compounds. Amongst those materials, co-crystals are the most complicated to categorize. The modern definition of a co-crystal arises from a scientific debate (Desiraju, 2003 ▸; Dunitz, 2003 ▸; Bond, 2007 ▸) on both the appropriateness of the word and what it actually stands for.

The proposed alternatives for the term co-crystal, including ‘molecular complex’, are ambiguous and do not include some of the more complicated cases. An example can be co-crystals formed by one or more liquid substrates not intended as solvents, *e.g.* pyridine:formic acid co-crystal (Wiechert & Mootz, 1999 ▸) or a series of co-crystals with pyrazine and *n*-alkyl­carb­oxy­lic acids where some co-formers are solid and some liquid (Bond, 2003 ▸, 2006 ▸). The US Food and Drug Administration (FDA) formulated their own classification of pharmaceutical co-crystals as ‘dissociable active pharmaceutical ingredient (API)-excipient molecular complexes’ [US Department of Health & Human Services, Food & Drug Administration Center for Drug Evaluation & Research (CDER), 2016 ▸]. This forced a more uniform response from the scientific community.

In 2012, a broader definition was proposed, where co-crystals are ‘crystalline single-phase materials built of two or more molecular and/or ionic co-formers generally in a stoichiometric ratio’ (Aitipamula *et al.*, 2012 ▸). This includes some of the nontrivial solvate cases (Bond, 2003 ▸, 2006 ▸) or so called ionic co-crystals (Smith *et al.*, 2013 ▸; Braga *et al.*, 2010 ▸, 2012 ▸; Zaworotko *et al.*, 2013 ▸). Unfortunately it still does not give a clear distinction between a salt and a co-crystal (Childs *et al.*, 2007 ▸). At the same time, different authors added subclasses including solvated co-crystals, non-solvated salt co-crystals, solvated salt co-crystals and solvated salts.

Matters then get even more complicated when trying to define what salt co-crystals actually are. The term resembles ionic co-crystals and in some papers they are used interchangeably (Thaimattam *et al.*, 2008 ▸; Golovnev *et al.*, 2016 ▸), whereas in others there is a clear distinction based on the nature of the ionic component (Braga *et al.*, 2011 ▸). In the above-mentioned cases, there is one molecular component and one salt component present: an inorganic alkaline/alkaline earth metal salt (ionic co-crystal; Braga *et al.*, 2011 ▸) or salified API (salt co-crystal; Berry & Steed, 2017 ▸). A few questions arise: how do we classify systems with more than three organic components: both ionic and molecular and/or containing solvents? How do we distinguish by name a salt co-crystal with one ionic component from those with two or more ionic components of the same type? There have been many further attempts for a coherent classification (Aitipamula *et al.*, 2012 ▸; Grothe *et al.*, 2016 ▸); however, the problem of formulation still remains.

**Figure 1:**
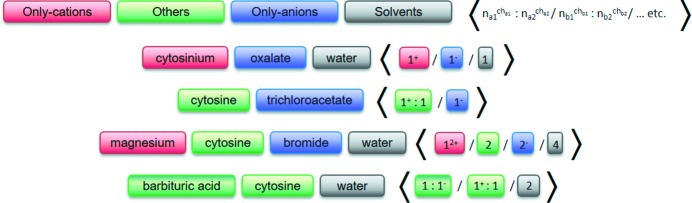
Graphical representations of new nomenclature formation; *n*_*a*1_ – stoichiometric index of component ‘*a*’ in ionic form ‘1’, ch_*a*1_ – charge of the form ‘*a*1’. The diagram is colour coded to show respective parts of the names.

In this manuscript, we would like to prove that all cases of multicomponent materials can be described by a coherent nomenclature showing what is actually inside the solid in question (Fig. 1 ▸). The classification of any multicomponent material should be decided separately for each case. This concept was devised during analysis of a multicomponent crystal containing one cytosine molecule, one cytosinium cation, one barbituric acid molecule, a barbiturate anion and two water molecules (abbreviated here as barcyt). As this new crystal structure is not the only example of a complicated system containing cytosine, we have used this molecule as a target for a CSD search for different examples of salt and co-crystals. In multicomponent materials cytosine can be found both in molecular and ionic forms separately or together as cytosine–cytosinium (CCH^+^) base pairs. Chemically cytosine is a nucleobase which plays a key role in the formation of heteromeric aggregates in DNA/RNA through N—H⋯N and N—H⋯O hydrogen bonds (Benabou *et al.*, 2014 ▸; Liu & Balasubramanian, 2003 ▸). Examination of the CSD database revealed a rich chemistry of cytosine-based multicomponent solids. Amongst the examined structures we found simple solvates, salts, salt solvates, co-crystals, ionic co-crystal solvates, salt co-crystals, salt co-crystal solvates and other cases more difficult to classify. This variety of crystal structures makes cytosine an ideal target molecule for the formulation and validation of a general set of rules for the nomenclature of multicomponent materials.

## 2. Nomenclature set of rules   

The naming scheme should be (in order of importance): systematic, unambiguous, unified for all known co-crystals, flexible to include new cases, precise, concise and easy. In agreement with these guidelines the following rules for naming co-crystals were set:

(1) The name consists of a list of components (separated by a space) followed by a composition and charge descriptor enclosed in angle brackets (〈〉). The list of components includes four groups: (*a*) only cationic components, (*b*) components with neutral form present in the structure no matter if an other ionic form is present, (*c*) only anionic components, (*d*) solvent molecules. Each of the four groups is sorted in alphabetical order.

(2) Each co-crystal component is mentioned only once regardless of the number of its ionic forms (protonated or deprotonated) present in the structure. The name of the neutral form is used in group (*b*). For groups (*a*) and (*c*), the name of the proper form is used following the rules of the respective cation or anion nomenclature.

(3) The composition and charge descriptor in angle brackets (〈〉) consists of fields separated by solidus character (/), the number of fields is equal to the number of listed components. Each field contains stoichiometric indices (as mutually prime numbers) of every ionic form of the respective component per formula unit. The charge is indicated in superscript following each stoichiometric index, charge equal 0 is omitted, charge +1 and −1 are indicated only with + or −, respectively. If more than one ionic form of one component is present their indices are separated with a colon (:). All ionic forms for a particular component are described in order from the most positive to the most negative.

These three rules, however simple, still require some further explanation. Naming every component only once allows the name to be kept as short as possible while still retaining all the chemical information. Using long names is not only difficult but impractical. On the other hand, splitting the components into the four mentioned groups seems necessary in order to keep the name in accordance with concurrent naming schemes of other compounds.

Due to this simple alteration the cations (often with names ending with -ium) will always be mentioned first and the anions (often with names ending with -ate or -ide) last (apart from solvents), keeping the name logical and consistent. Note that for co-crystals group (*a*), (*c*) and (*d*) may be absent for some compounds (see Fig. 1 ▸); however, group (*b*) will always be there due to requirements of the co-crystal definition. On the other hand, group (*b*) will not occur for salts and group (*d*) will always be present for solvates. The decision on whether a component in the co-crystal structure is a solvent molecule is not always trivial. Therefore, each particular case should be analysed separately and a choice should be made as to whether to put a component in group (*d*) as a solvent or in group (*b*). We propose the following distinction: the component should be regarded as a solvent if these conditions are met simultaneously: (i) it exists in the structure only in neutral state, no other ionic form is present; (ii) the component exists in standard conditions in liquid form and (iii) it can be regarded as a common solvent. This differentiation is arbitrary and artificial but this definition of a solvent can be relatively easy incorporated into a computer code for automatic naming routines. To date, angle brackets have not been used either in chemical formulae or chemical names. The use of angle brackets will allow the name of solid phase of a co-crystal to be easily distinguished from any other type of compound. The solidus (/) is used in names of formal addition compounds to separate the stoichiometric indices of the individual components [*e.g.* boron trifluoride—water (1/2); Connelly *et al.*, 2005]. Here a similar application is proposed. The colon is used so far in names of polynuclear coordination compounds, boron compounds and in nodal descriptors of inorganic chains and rings (Connelly *et al.*, 2005 ▸). Presented use of the colon does not interfere with the above-mentioned applications. Both solidus and colon are commonly used to describe ratio, therefore their use seems natural.

Although we find very little use for a full name of a multicomponent solid in spoken language, the need to read it may occur occasionally. In such a case the punctuation should not be vocalized and the pauses and proper intonation should be used to clearly and correctly list all the indices – longer pause for solidus and shorter for colon.

Please note that all the multiplicative prefixes and oxidation state descriptors should be omitted before the descriptor, as all the information they carry will be shown in the brackets. The only remaining information that is not redundant is the optional poly prefix for polymeric coordination compounds (the nature of the polymer will be shown in the proper description of the component) and non-obvious oxidation states of some central atoms.

## 3. Testing of the nomenclature – cytosine-containing crystal structures   

The application of new nomenclature to the dihydrate adduct of cytosine barbiturate with barbituric acid and cytosine (barcyt, CCDC 1835517, crystal structure data presented in the supporting information) yields ‘barbituric acid cytosine water 〈1:1^−^/1^+^:1/2〉’. This is a simple, clear and concise way of describing this salt co-crystal solvate by giving the exact amount of components and describing their character. To make sure that the above-mentioned rules apply to a vast number of multicomponent solids we have tested the new nomenclature on 67 cytosine-containing multicomponent crystals taken from the CSD (Table S2). Those 67 test cases were selected using different and sometimes broad definitions of a co-crystal. For the sake of comparison, the discussed materials were further divided into seven categories: co-crystals, co-crystal solvates, salt co-crystals, salt co-crystal solvates, ionic co-crystal solvates, salts, salt solvates and other cases difficult to classify. The selected representatives of each category that facilitated the formulation of the final set of rules are presented in Table 1 ▸. The name reported in the CSD and the new name according to the proposed nomenclature were added as separate columns. Note that all the recalled crystal structures were reviewed in detail for the purpose of this work. The new name reflects the actual composition of the crystal, which for some cases was found to be slightly different than the reported name initially suggested. Additionally, the simplified formula, which allows a quick examination of the amount of different components presented in a particular material and CSD refcode were added. The variety of co-crystals presented in Table 1 ▸ emphasizes the simplicity of our approach. The target molecule, a co-former and all solvent molecules with their stoichiometry are mentioned in the name. In the most typical co-crystal there are two components and then the used name usually reflects the composition of the crystal such as in the case of cytosine 1,10-phenanthroline co-crystal (PUJWUO). For salt co-crystals and salt co-crystal solvates the reported names could be very confusing. This becomes even more complicated when the number of molecules of the components is not the same. The proposed nomenclature does not require the usage of a complicated name and thus prevents making a mistake with the amount of the reported ions and molecules as in the case of EPAMAK structure. The new name, cytosine 4-hy­droxy­benzoic acid water 〈1^+^:1/2:1^−^/2〉, clearly states the amount and the charge of each component. Moreover both systematic and common names can be used: *e.g.* cytosine or 4-amino­pyrimidin-2(1*H*)-one. A distinction between two ionic co-crystal solvates built from the same components although with different stoichiometry is straightforward using the new nomenclature. In fact two structures with refcodes ACITIQ and ACITOW will have the same core name, sodium cytosine perchlorate water, as the type of the components are the same; however the different stoichiometry will be obvious by looking at the 〈3^+^/3/3^−^/4〉 and 〈1^+^/1/1^−^/1〉 descriptors. In the case of ACITOW the initial indices in 〈3^+^/3/3^−^/3〉 were simplified leading to 〈1^+^/1/1^−^/1〉. In Table 1 ▸ there are also included non-traditional, to some extent problematic, cases of co-crystals. They include materials built of complex ions, cytosine molecules and/or anions playing a role of co-formers, not coordinated to the metal centre (IVORUG, MASXIQ). The new nomenclature also applies to polymeric structures as shown for 1D coordination polymer with cytosine connected through weak interactions to the polymeric chains (PEDYEC). The polymeric part (precisely the monomeric unit) is treated as one component and cytosine is the second one: poly[di­aquabis­(μ_2_-chloro)­cadmium] cytosine 〈1/2〉. Optionally, the dimensionality of the polymer may be indicated in the prefix [poly(1D)-, poly(2D)-, poly(3D)-] or the kappa convention may be used to specify which central atoms are bridged {*e.g.* poly[di­aqua-bis­(μ_2_-chloro-1:1′κ^2^Cl)cadmium] cytosine 〈1/2〉, here 1′ denotes cadmium atom from subsequent monomeric unit}. Another problematic situation occurs for the cases where cytosine plays a double role as a ligand and co-former (MAZGID). The organic co-former is treated as a separate entity from a complex ion, that contains cytosine as a ligand: *trans*-tetra­aquabis­(cytosine-O)manganese(II) cytosine per­chlorate water 〈1^2+^/2/2^−^/2〉. The new name contains all the information relevant to the clarity of the crystal composition, *i.e.* type and amount of components and their formal charge. This information is not easily deduced from the name reported in the CSD. It can also be used for nonstoichiometric compounds assuming normalization of stoichiometric indices, *e.g.* to values within the range 〈0;1〉. The cytosine-based co-crystals also show that the new nomenclature can be applied to systems containing quasi-symmetrical hydrogen bonding. This is the case with CCH^+^ base pairs, where one of the molecules is treated as an ion whereas the other should be neutral. However, we can treat such material as nonstoichiometric if the non-equal ratio of sites occupancy factors can be precisely determined (*e.g.* neutron diffraction experiements).

Please note that the proposed nomenclature was not formulated in order to replace the systematic name completely but to simplify the usage of often complicated names of multicomponent materials. Nowadays it is no longer a common practice to give the systematic name of a co-crystal in a manuscript; neither it is used in spoken language and thus the systematic names can be often found only in labels and CSD entries. Moreover, the names reported in the CSD are not systematized in any way. They use both common and systematic names of co-formers and, because of their complexity, often have errors in the composition. Usage of our naming scheme and incorporating it to the CSD would greatly improve the search for multicomponent materials. The naming process could be automatic, taking into account the simple and systematic rules of presented nomenclature as well as the advances in development of naming subroutines for organic and inorganic compounds.

## 4. Testing of the nomenclature – other complicated cases   

In this section we will cover other cases of multicomponent materials not containing cytosine but contributing to the understanding of proposed rules of chemical naming. This includes the materials containing mixed valence ions. The vast majority of such compounds would exhibit different coordination of atoms with different valence state and then, those two entities should be treated as two separate coordination ions. However, for the few cases where the only difference between coordination entities is the valence state of the metal or different protonation state of the ligand (not influencing the connectivity inside the complex), both forms can be regarded as the same component with different ionic states. As an example, the structure with refcode DTHCUA named bis­[bis­(2,5-di­thia­hexane-*S*,*S*′)copper(I)] bis­(2,5-di­thia­hexane-*S*,*S*′)copper(II) tetraperchlorate can be named bis­(2,5-di­thia­hexane-*S*,*S*′)copper perchlorate 〈2^+^:1^2+^/4^−^〉. Species with multiple degrees of protonation are also covered by the new nomenclature. An example of this is the crystal structure with CSD refcode JALKUF, which contains five cations of sodium and two citrate anions: one with 3^−^ and other with 2^−^ charge. The systematic name reported in the CSD is penta­kis­(sodium) hydrogen dicitrate, whereas the new proposed name is sodium citrate 〈5^+^/1^2−^:1^3−^〉. Protonated solvent molecules should be treated as any other cations, whereas the presence of both neutral and protonated solvent molecules forces their assignment to the second group of components (see ‘Others’, Fig. 1 ▸). An example of this is the crystal structure with refcode BALRUB and the reported name in CSD of ammonium chloro­anilate oxonium monohydrate can be renamed ammonium water chloro­anilate 〈1^+^/1^+^:1/1^2−^〉. In this crystal structure, water molecules coexist with oxonium ions and as such we report this component only once as a neutral molecule. Water is not placed in the solvent section of the name as the oxonium ion should be treated as a regular cationic component of this structure and as such the name water appears after the purely cationic form.

## 5. Conclusions   

We have presented a uniform and concise nomenclature for multifunctional materials, including co-crystals, salt co-crystals, salt co-crystal solvates, ionic co-crystals, ionic co-crystal solvates, salts, salt solvates and other cases difficult to classify. We have proven that the proposed nomenclature perfectly deals with extraordinary cases of multicomponent materials containing multiple building blocks with different charges and clarifies the nontrivial cases of co-formers being in a liquid state prior to co-crystal formation. The presented approach is our contribution to the ongoing scientific debate on the definition of a co-crystal. In fact the proposed nomenclature proves that the general classification of co-crystals is not as important as showing what kind of species are actually hidden in the crystal.

## Supplementary Material

Structure factors: contains datablock(s) I. DOI: 10.1107/S2052520618015858/px5009Isup2.hkl

Crystal structure: contains datablock(s) I. DOI: 10.1107/S2052520618015858/px5009sup1.cif

Supporting information, Figs. S1, S2 and Tables S1, S2. DOI: 10.1107/S2052520618015858/px5009sup3.pdf

CCDC reference: 1835517

## Figures and Tables

**Table 1:** An overview of multicomponent crystals containing cytosine with the proposed, new nomenclature scheme The letter A denotes any co-former or its ionic form.

Reported name	New name	Simplified formula	CSD refcode†
Co-crystal			
Cytosine 1,10-phenanthroline	Cytosine 1,10-phenanthroline 〈1/1〉	CytA	PUJWUO^*a*^
2-Amino-6-phenyl­pyrimidin-4(3*H*)-one 4-amino­pyrimidin-2(1*H*)-one	2-Amino-6-phenyl­pyrimidin-4(3*H*)-one 4-amino­pyrimidin-2(1*H*)-one 〈1/1〉	CytA	QOBCOB^*b*^

Co-crystal solvate			
Cytosine 5-fluoro­uracil monohydrate	Cytosine 5-fluoro­uracil water 〈1/1/1〉	CytA·H_2_O	CYTFUR01^*c*^
2-Amino-5-iso­propyl-6-methyl­pyrimidin-4(3*H*)-one 4-amino­pyrimidin-2(1*H*)-one monohydrate	2-Amino-5-iso­propyl-6-methyl­pyrimidin-4(3*H*)-one 4-amino­pyrimidin-2(1*H*)-one water 〈1/1/1〉	CytA·H_2_O	QOBCER^*b*^

Ionic co-crystal solvate			
*catena*-[Tris­(μ_3_-cytosine-*O*,*O*,*O*)tris­(μ_2_-aqua)(μ_2_-perchlorato-*O*,*O*′)aquatrisodium diperchlorate]	Sodium cytosine perchlorate water 〈3^+^/3/3^−^/4〉	(Na^+^)_3_Cyt_3_(ClO_4_^−^)_3_·4H_2_O	ACITIQ^*d*^
*catena*-[Tris­(μ_3_-cytosine-*O*,*O*,*O*)tris­(μ_2_-aqua)(μ_2_-perchlorato-*O*,*O*′)(perchlorato-*O*)trisodium perchlorate]	Sodium cytosine perchlorate water 〈1^+^/1/1^−^/1〉	(Na^+^)_3_Cyt_3_(ClO_4_^−^)_3_·3H_2_O	ACITOW^*d*^
Bis[4-amino­pyrimidin-2(1*H*)-one]tetraaqua­magnesium bis­(bromide)	Magnesium 4-amino­pyrimidin-2(1*H*)-one bromide water 〈1^2+^/2/2^−^/4〉	Mg^2+^Cyt_2_(Br^−^)_2_·4H_2_O	NALFIS^*e*^

Salt co-crystal			
Dicytosine tri­chloro­acetate	Cytosine tri­chloro­acetate 〈1^+^:1/1^−^〉	CytH^+^CytA^−^	GITYEN^*f*^
Cytosinium benzoate cytosine benzoic acid	Benzoic acid cytosine 〈1:1^−^/1^+^:1〉	CytH^+^Cyt(AH)A^−^	TAZWUN^*g*^

Salt co-crystal solvate			
Cytosinium cytosine hydrogen maleate	Cytosine maleate water 〈1^+^:1/1^−^/1〉	CytH^+^CytAH^−^·H_2_O	DUJCAN^*h*^
Cytosinium 4-hy­droxy­benzoate cytosine bis­(4-hy­droxy­benzoic acid) cytosine dihydrate	Cytosine 4-hy­droxy­benzoic acid water 〈1^+^:1/2:1^−^/2〉	CytH^+^Cyt(AH)_2_A^−^·2H_2_O	EPAMAK^*i*^
Cytosine cytosinium di­cyano­{4-[di­cyano­(meth­oxy)­methyl]­phenyl}­methanide methanol solvate	Cytosine di­cyano­{4-[di­cyano­(meth­oxy)­methyl]­phenyl}­methanide methanol 〈1^+^:1/1^−^/1〉	CytH^+^CytA^−^·MeOH	LIWSOA^*j*^

Salt/salt solvate			
Cytosinium hydrogen sulfate	Cytosinium sulfate 〈1^+^/1^−^〉	CytH^+^A^−^	CUKCUH^*k*^
Cytosinium hydrogen oxalate monohydrate	Cytosinium hydrogen oxalate water 〈1^+^/1^−^/1〉	CytH^+^A^−^·H_2_O	CASCIJ^*l*^
Bis­(6-amino-2-oxo-2,3-di­hydro­pyrimidin-1-ium) naphthalene-1,5-di­sulfonate	Bis­(6-amino-2-oxo-2,3-di­hydro­pyrimidin-1-ium) naphthalene-1,5-di­sulfonate 〈2^+^/1^2−^〉	(CytH^+^)_2_A^2−^	PATYAN^*m*^
Cytosinium diaqua(nitrilo­tri­acetato-*N*,*O*,*O*′,*O*′′)nickel(II) dihydrate	Cytosinium di­aqua-(nitrilo­tri­acetato-*N*,*O*,*O*′,*O*′′)nickelate(II) water 〈1^+^/1^−^/2〉	CytH^+^A^−^·2H_2_O	QOCTOR^*n*^

Other nontrivial cases of co-crystals/multicomponent solids			
[1,2-Bis­(amidino-*O*-methyl­urea)ethane]­copper(II) bis­(cytosine) bis­(tetra­fluoro­borate)	[1,2-Bis­(amidino-*O*-methyl­urea)ethane]­copper(II) cytosine tetra­fluoro­borate 〈1^2+^/2/2^−^〉	[Cu*L*]^2+^Cyt_2_([BF_4_]^−^)_2_	IVORUG^*o*^
Bis­(cytosinium) tetra­chlorocobalt(II) bis­(cytosine)	Cytosine tetra­chloro­cobaltate(II) 〈2^+^:2/1^2−^〉	(CytH^+^)_2_Cyt_2_[CoCl_4_]^2−^	MASXIQ^*p*^
*catena*-[Di­aqua­bis­(μ_2_-chloro)dicadmium bis­(cytosine)]	Poly[di­aquabis­(μ_2_-chloro)cadmium] cytosine 〈1/2〉	[Cd(μ_2_-Cl)_2_(H_2_O)_2_]_*n*_Cyt_2*n*_	PEDYEC^*q*^
*catena*-[Penta­kis­(μ_2_-aquo)tri­aquabis­(cytosine)-(μ_2_-decavanadate)tris­odium tris­(6-amino-2-oxo-2,3-di­hydro­pyrimidin-1-ium)cytosine dihydrate]	Sodium cytosine decavanadate water 〈3^+^/3^+^:3/1^6−^/10〉	(Na^+^)_3_(CytH^+^)_3_Cyt_3_(V_10_O_28_)^6−^·10H_2_O	BUPMAB^*r*^
*trans*-Tetraaquabis­(cytosine-*O*)manganese(II) diperchlorate cytosine solvate dihydrate	*trans*-Tetra­aquabis­(cytosine-*O*)manganese(II) cytosine perchlorate water 〈1^2+^/2/2^−^/2〉	[Mn(Cyt)_2_(H_2_O)_4_]^2+^Cyt_2_(ClO_4_^−^)_2_·2H_2_O	MAZGID^*s*^

† References: (*a*) Hoxha *et al.* (2015 ▸); (*b*) Radhakrishnan *et al.* (2014 ▸); (*c*) Portalone & Colapietro (2007 ▸); (*d*) Armentano *et al.* (2006 ▸); (*e*) Marino *et al.* (2016 ▸); (*f*) Gdaniec *et al.* (1988 ▸); (*g*) Perumalla *et al.* (2005 ▸); (*h*) Benali-Cherif *et al.* (2009 ▸); (*i*) Sridhar & Ravikumar (2010 ▸); (*j*) Murata *et al.* (2008 ▸); (*k*) Bensegueni *et al.* (2009 ▸); (*l*) Bouchouit *et al.* (2005 ▸); (*m*)Singh *et al.* (2017 ▸); (*n*) Salam & Aoki (2000 ▸); (*o*) Suksangpanya *et al.* (2004 ▸); (*p*) Thomas-Gipson *et al.* (2017 ▸); (*q*) De Munno *et al.* (1993 ▸); (*r*) Bosnjakovic-Pavlovic *et al.* (2009 ▸); (*s*) De Munno *et al.* (2000 ▸).
