# Supplementary material for: A proposal for coherent nomenclature of multicomponent crystals
Source: Acta Crystallogr B Struct Sci Cryst Eng Mater. 2019 Jan 23;75(Pt 1):53–8. doi: 10.1107/S2052520618015858 (PMC6457040; doi:10.1107/S2052520618015858)
Supplement: Supplementary file 3 [file b-75-00053-sup3.pdf]

STRUCTURAL SCIENCE
CRYSTAL ENGINEERING
MATERIALS

Volume 75 (2019)

Supporting information for article:

A proposal for coherent nomenclature of multi-component crystals

Marlena Gryl, Marcin Kozieł and Katarzyna M. Stadnicka

S1. Cocrystallisation of barcyt

The dihydrate adduct of cytosine barbiturate with barbituric acid and cytosine (**barcyt**) was obtained by mixing aqueous solutions of barbituric acid and cytosine in equimolar ratio at temperature *ca.* 343K. The product was recrystallized from the mixture of ethanol and water in the ratio 2:1. The structure of barcyt follows the symmetry of space group $P\bar{1}$. Contents of the asymmetric unit with atom numbering scheme is presented in Figure S1, whereas the packing of the structural components can be seen in Figure S2. Details of the data collection and the refinement procedure are presented in Table S1.

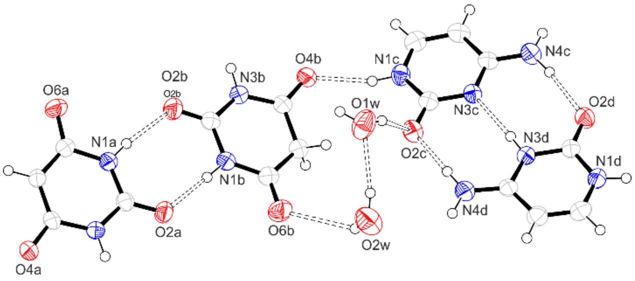

Figure S1 Asymmetric unit of barcyt with the atom numbering scheme. Displacement ellipsoids are drawn at 50% probability level.

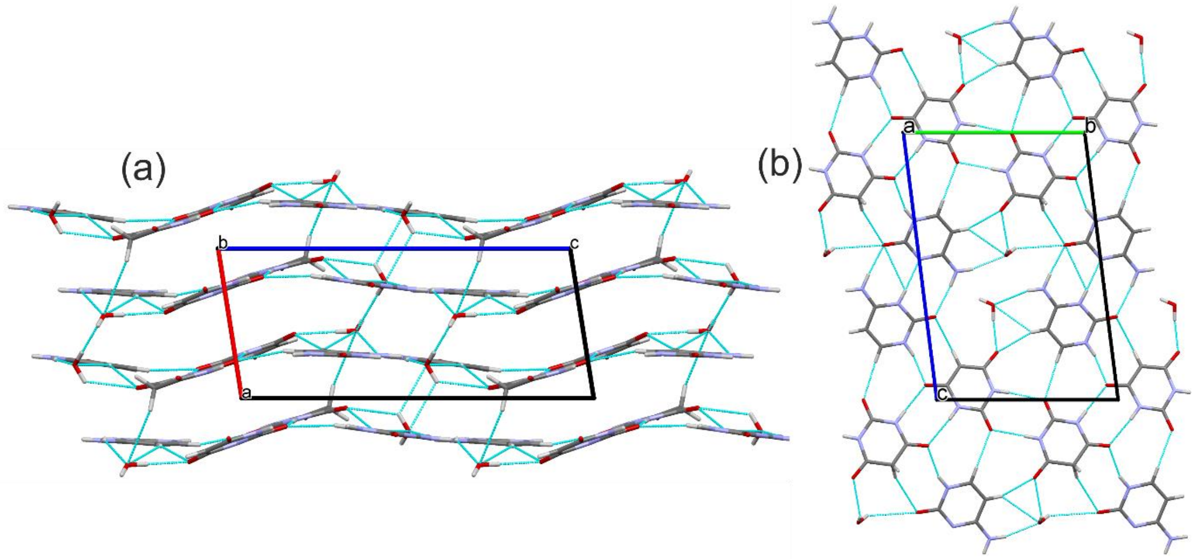

Figure S2 a) Packing of the **barcyt** structure components a) viewed along [010] b) the layer 0≤a≤0.25 viewed along [100].

Table S1 Crystal data and structure refinement details for **barcyt**.

Identification code	**barcyt**
Empirical formula	$C_{16}H_{22}N_{10}O_{10}$
Formula weight	514.44
Temperature	293(2) K
Wavelength	0.71073 Å
Crystal system	Triclinic
Space group	$P\bar{1}$
Unit cell dimensions	a = 6.7486(3) Å α= 81.293(3)°
	b = 10.5419(4) Å β= 79.732(2)°
	c = 15.6580(7)Å γ= 79.974(3)°
Volume	1071.02(8) $Å^3$
Z	2
Density (calculated)	1.595 g/cm^3
Absorption coefficient	0.134 mm^{-1}
F(000)	536
Crystal size	0.24 x 0.13 x 0.10 mm
Diffractometer	Nonius KappaCCD*
Theta range for data collection	3.12 to 25.50°
Index ranges	-7≤h≤8, -12≤k≤12, 0≤l≤18
Reflections collected	8425
Independent reflections	3947 [R(int) = 0.0770]
Completeness to theta = 25.50°	98.9 %
Reflections with I>2sigma(I)	2509
Absorption correction	Multi-scan**
Max. and min. transmission	0.987and 0.969
Refinement method	Full-matrix least-squares on F^2
Data / restraints / parameters	3947 / 17 / 370
Goodness-of-fit on F2	1.024
Final R indices [I>2sigma(I)]	R1 = 0.0449, wR2 = 0.0984
R indices (all data)	R1 = 0.0861, wR2 = 0.1110
Largest diff. peak and hole	0.235 and -0.203 $eÅ^{-3}$

* Nonius (1998). *COLLECT*. Nonius BV, Delft, The Netherlands.

** DENZO – SMN (Otwinowski & Minor, 1997)

Table S2 New nomenclature for cytosine multi-component materials.

reported name	new name	simplified formula	refcode
co-crystal			
Chrysin cytosine	Chrysin cytosine $\langle 1/1\rangle$	CytA	IYIWEU
Cytosine 1,10-phenanthroline	Cytosine 1,10-phenanthroline $\langle 1/1\rangle$	CytA	PUJWUO
2-amino-6-phenylpyrimidin-4(3H)-one 4-aminopyrimidin-2(1H)-one	2-amino-6-phenylpyrimidin-4(3H)-one 4-aminopyrimidin-2(1H)-one $\langle 1/1\rangle$	CytA	QOBCOB
co-crystal solvate			
cytosine 5-fluorouracil monohydrate	cytosine 5-fluorouracil water $\langle 1/1/1\rangle$	$CytA\cdot H_2O$	CYTFUR01
2-amino-5-isopropyl-6-methylpyrimidin-4(3H)-one 4-aminopyrimidin-2(1H)-one monohydrate	2-amino-5-isopropyl-6-methylpyrimidin-4(3H)-one 4-aminopyrimidin-2(1H)-one water $\langle 1/1/1\rangle$	$CytA\cdot H_2O$	QOBCER
ionic co-crystal solvate			
catena-(tris(μ_3-Cytosine-O,O,O)-tris(μ_2-aqua)-(μ_2-perchlorato-O,O')-aqua-tri-sodium diperchlorate)	sodium cytosine perchlorate water$\langle 3^{+}/3/3^{-}/4\rangle$	$(Na^{+})_3Cyt_3(ClO_4^{-})_3\cdot 4H_2O$	ACITIQ
catena-(tris(μ_3-Cytosine-O,O,O)-tris(μ_2-aqua)-(μ_2-perchlorato-O,O')-(perchlorato-O)-tri-sodium perchlorate)	sodium cytosine perchlorate water$\langle 1^{+}/1/1^{-}/1\rangle$	$(Na^{+})_3Cyt_3(ClO_4^{-})_3\cdot 3H_2O$	ACITOW
Cytosine calcium chloride hydrate	calcium cytosine chloride water $\langle 1^{2+}/1/2^{-}/1\rangle$	$Ca^{2+}Cyt(Cl^{-})_2\cdot H_2O$	CYTSCA
bis(4-aminopyrimidin-2(1H)-one)-tetraaqua-magnesium bis(bromide)	magnesium 4-aminopyrimidin-2(1H)-one bromide water$\langle 1^{2+}/2/2^{-}/4\rangle$	$Mg^{2+}Cyt_2(Br^{-})_2\cdot 4H_2O$	NALFIS
bis(4-aminopyrimidin-2(1H)-one)-tetraaqua-magnesium bis(chloride)	magnesium 4-aminopyrimidin-2(1H)-one chloride water$\langle 1^{2+}/2/2^{-}/4\rangle$	$Mg^{2+}Cyt_2(Cl^{-})_2\cdot 4H_2O$	NALFOY
tetrakis(4-aminopyrimidin-2(1H)-one)-diaqua-magnesium bis(bromide)	magnesium 4-aminopyrimidin-2(1H)-one bromide water$\langle 1^{2+}/4/2^{-}/2\rangle$	$Mg^{2+}Cyt_4(Br^{-})_2\cdot 2H_2O$	NALFUE
tetrakis(4-aminopyrimidin-2(1H)-one)-diaqua-magnesium bis(chloride)	magnesium 4-aminopyrimidin-2(1H)-one chloride water $\langle 1^{2+}/4/2^{-}/2\rangle$	$Mg^{2+}Cyt_4(Cl^{-})_2\cdot 2H_2O$	NALGAL
Tetra-aqua-bis(cytosine)-magnesium diperchlorate cytosine solvate dihydrate	magnesium cytosine perchlorate water $\langle 1^{2+}/4/2^{-}/6\rangle$	$Mg^{2+}Cyt_4(ClO_4^{-})_2\cdot 6H_2O$	RIVCII
bis(cytosine)-tetraaqua-calcium bis(cytosine) diperchlorate dihydrate	calcium cytosine perchlorate water $\langle 1^{2+}/4/2^{-}/6\rangle$	$Ca^{2+}Cyt_4(ClO_4^{-})_2\cdot 6H_2O$	XOMKAN
bis(μ-cytosine)-tetraaqua-bis(perchlorato-O)-bis(perchlorato-O,O')-di-calcium	calcium cytosine perchlorate water $\langle 1^{2+}/1/2^{-}/2\rangle$	$Ca^{2+}Cyt(ClO_4^{-})_2\cdot 2H_2O$	XOMKER
bis(μ-cytosine)-tetraaqua-dichloro-bis(cytosine)-di-calcium dichloride	calcium cytosine chloride water $\langle 1^{2+}/2/2^{-}/2\rangle$	$Ca^{2+}Cyt_2(Cl^{-})_2\cdot 2H_2O$	XOMKIV
salt co-crystal			
Cytosinium cytosine tetrafluoroborate	cytosine tetrafluoroborate $\langle 1^{+}:1/1^{-}\rangle$	$CytH^{+}Cyt[BF_4]^{-}$	ACITEM
cytosine malonic acid	cytosine malonate $\langle 1^{+}:1/1^{-}\rangle$	$CytH^{+}Cyt(AH^{-})$	CUVDON
cytosine succinic acid	cytosine succinate $\langle 1^{+}:1/1^{-}\rangle$	$CytH^{+}Cyt(AH^{-})$	CUVDUT
Dicytosine trichloroacetate	cytosine trichloroacetate $\langle 1^{+}:1/1^{-}\rangle$	$CytH^{+}CytA^{-}$	GITYEN
Cytosinium cytosine 2,5-diethyl-7,7,8,8-tetracyanoquinodimethane	cytosine 2,5-diethyl-7,7,8,8-tetracyanoquinodimethanate $\langle 1^{+}:1/1^{\bullet -}\rangle$	$CytH^{+}CytA^{\bullet -}$	LIWSIU
Cytosinium cytosine 7,7,8,8-tetracyanoquinodimethane	cytosine 7,7,8,8-tetracyanoquinodimethanate $\langle 1^{+}:1/1^{\bullet -}\rangle$	$CytH^{+}CytA^{\bullet -}$	ODICOU
bis(Cytosine) adipic acid	cytosine adipate $\langle 1^{+}:1/1^{-}\rangle$	$CytH^{+}Cyt(AH^{-})$	OYEREQ01
cytosinium benzoate cytosine benzoic acid	benzoic acid cytosine $\langle 1:1^{-}/1^{+}:1\rangle$	$CytH^{+}Cyt(AH)A^{-}$	TAZWUN

Cytosine cytosinium tribromoacetate	cytosine tribromoacetate ⟨1^+:1/1^-⟩	$CytH^+CytA^-$	VISVIE
2-Oxo-2,3-dihydropyrimidin-4(1H)-iminium 4-aminopyrimidin-2(1H)-one 5-chlorothiophene-2-carboxylate	4-aminopyrimidin-2(1H)-one chlorothiophene-2-carboxylate ⟨1^+:1/1^-⟩	$CytH^+CytA^-$	XOMZUV
2-Oxo-2,3-dihydropyrimidin-4(1H)-iminium 2-((2,3-dimethylphenyl)amino)benzoate 4-aminopyrimidin-2(1H)-one	4-aminopyrimidin-2(1H)-one 2-((2,3-dimethylphenyl)amino)benzoate ⟨1^+:1/1^-⟩	$CytH^+CytA^-$	ZAZGEO
bis(Hemicytosinium) saccharinate	cytosine saccharinate ⟨1^+:1/1^-⟩	$CytH^+CytA^-$	ZEYKAR
bis(Hemicytosinium) 2,6-dinitrophenolate	cytosine 2,6-dinitrophenolate ⟨1^+:1/1^-⟩	$CytH^+CytA^-$	ZEYKEV
bis(Cytosine) fumaric acid	cytosine fumarate ⟨1^+:1/1^-⟩	$CytH^+CytAH^-$	ZEYNOI
salt co-crystal solvate			
Cytosinium hydrogen adamantane-1,3-dicarboxylate cytosine monohydrate	cytosine adamantane-1,3-dicarboxylate water ⟨1^+:1/1^-/1⟩	$CytH^+CytAH^- \cdot H_2O$	ZEYLEW
Cytosinium dihydrogen citrate bis(cytosine) monohydrate	cytosine citrate water ⟨3^+:3/1^-/1^{2-}/2⟩	$CytH^+Cyt(AH_2^-)\cdot H_2O$	OYERIU
cytosine oxalic acid hemihydrate	cytosine oxalate water ⟨2^+:2/1^{2-}/2⟩	$(CytH^+)_2Cyt_2A^{2-}\cdot 2H_2O$	CUVDIH01
bis(Cytosine) μ-resorcylic acid complex monohydrate	cytosine γ-resorcylate water ⟨1^+:1/1^-/1⟩	$CytH^+CytA^-\cdot H_2O$	CYTRES10
2-oxo-2,3-dihydro-4(1H)-aminopyrimidinium 3-oxido-6-oxo-1,6-dihydro-pyridazine 2-oxo-1,2-dihydro-4-aminopyrimidine 6-hydroxy-3(2H)-pyridazinone dihydrate	3-oxido-6-oxo-1,6-dihydro-pyridazine 2-oxo-1,2-dihydro-4-aminopyrimidine water ⟨1:1^-/1^+:1/2⟩	$CytH^+Cyt(AH)A^- \cdot 2H_2O$	DOTGOJ
Cytosinium cytosine hydrogen maleate	cytosine maleate water ⟨1^+:1/1^-/1⟩	$CytH^+CytAH^-\cdot H_2O$	DUJCAN
Cytosinium isonicotinate cytosine dihydrate	cytosine isonicotinate water ⟨1^+:1/1^-/2⟩	$CytH^+CytA^-\cdot 2H_2O$	DUZNOC
cytosinium 4-nitrobenzoate cytosine monohydrate	cytosine 4-nitrobenzoate water ⟨1^+:1/1^-/1⟩	$CytH^+CytA^-\cdot H_2O$	EFUNUP
Cytosinium 4-hydroxybenzoate cytosine bis(4-hydroxybenzoic acid) cytosine dihydrate	cytosine 4-hydroxybenzoic acid water ⟨1^+:1/2:1^-/2⟩	$CytH^+Cyt(AH)_2A^- \cdot 2H_2O$	EPAMAK
6-Amino-2-oxo-2,3-dihydropyrimidin-1-ium 5-nitro-2,4-dioxo-3,4-dihydro-2H-pyrimidin-1-ide 5-nitropyrimidine-2,4(1H,3H)-dione 4-aminopyrimidin-2(1H)-one dihydrate	4-aminopyrimidin-2(1H)-one 5-nitropyrimidine-2,4(1H,3H)-dione water ⟨1^+:1/1:1^-/2⟩	$CytH^+Cyt(AH)A^- \cdot 2H_2O$	GATMOF
Cytosine cytosinium dicyano(2,3,5,6-tetrafluoro-4-(dicyano(methoxy)methyl)phenyl)methanide monohydrate	cytosine dicyano(2,3,5,6-tetrafluoro-4-(dicyano(methoxy)methyl)phenyl)methanide water ⟨1^+:1/1^-/1⟩	$CytH^+CytA^-\cdot H_2O$	KEVSAG
Cytosine cytosinium dicyano(4-(dicyano(methoxy)methyl)phenyl)methanide methanol solvate	cytosine dicyano(4-(dicyano(methoxy)methyl)phenyl)methanide methanol ⟨1^+:1/1^-/1⟩	$CytH^+CytA^-\cdot MeOH$	LIWSOA
Cytosinium sulfosalicylate cytosine monohydrate	cytosine sulfosalicylate water ⟨1^+:1/1^-/1⟩	$CytH^+CytA^-\cdot H_2O$	UWANAJ
Cytosinium 4-amino-2-hydroxybenzoate cytosine monohydrate	cytosine 4-amino-2-hydroxybenzoate water ⟨1^+:1/1^-/1⟩	$CytH^+CytA^-\cdot H_2O$	XICRIM
Cytosinium 4-hydroxyphenylacetate cytosine monohydrate	cytosine 4-hydroxyphenylacetate water ⟨1^+:1/1^-/1⟩	$CytH^+CytA^-\cdot H_2O$	ZEGFAU
Cytosinium 4-aminobenzoate cytosine monohydrate	cytosine 4-aminobenzoate water ⟨1^+:1/1^-/1⟩	$CytH^+CytA^-\cdot H_2O$	ZEGFEY
Cytosinium acetylenedicarboxylate cytosine monohydrate	cytosine acetylenedicarboxylate water ⟨2^+:2/1^{2-}/2⟩	$(CytH^+)_2Cyt_2A^{2-}\cdot 2H_2O$	ZEYLIA
Other, unclassified cases			
(1,2-bis(Amidino-O-methylurea)ethane)-copper(ii) bis(cytosine) bis(tetrafluoroborate)	(1,2-bis(Amidino-O-methylurea)ethane)-copper(II) cytosine tetrafluoroborate ⟨1^{2+}/2/2^-⟩	$[CuL]^{2+}Cyt_2([BF_4]^-)_2$	IVORUG
bis(cytosinium) tetrachloro-cobalt(ii) bis(cytosine)	cytosine tetrachloro-cobaltate(II) ⟨2^+:2/1^{2-}⟩	$(CytH^+)_2Cyt_2[CoCl_4]^{2-}$	MASXIQ

catena(Diaqua-bis((μ_2-chloro))-di-cadmium bis(cytosine))	poly-(diaqua-bis(μ_2-chloro)-cadmium) cytosine $\langle 1/2\rangle$	$[Cd(\mu_2\text{-}Cl)_2(H_2O)_2]_nCyt_{2n}$	PEDYEC
Cytosinium diaqua-(nitrilotriacetato-N,O,O',O'')-nickel(ii) cytosine dihydrate	cytosine diaqua-(nitrilotriacetato-N,O,O',O'')-nickelate(II) water $\langle 1^+:1/1^-/2\rangle$	$CytH^+Cyt[Ni(L)(H_2O)_2]^- \cdot 2H_2O$	QOCTUX
bis((1H,3H)-Cytosinium) aqua-bis(pyridine-2,6-dicarboxylato)-manganese(ii) cytosine solvate hexahydrate	cytosine aqua-bis(pyridine-2,6-dicarboxylato)-manganate(II) water $\langle 2^+:2/1^{2-}/6\rangle$	$(CytH^+)_2Cyt_2[MnL_2(H_2O)]^{2-}\cdot 6H_2O$	QUTJUL
tetrakis(2-oxo-2,3-dihydropyrimidin-4(1H)-iminium) bis(μ_5-selenito)-pentakis(μ_2-oxo)-decaoxo-penta-molybdenum cytosine solvate hexahydrate	4-aminopyrimidin-2(1H)-one bis(μ_5-selenito)-pentakis(μ_2-oxo)-decaoxo-penta-molybdate water $\langle 4^+:4/1^{4-}/6\rangle$	$(CytH^+)_4Cyt_4[Mo_5O_{21}Se_2]^{4-}\cdot 6H_2O$	TIZCUC
6-Amino-2-oxo-2,3-dihydropyrimidin-1-ium 6-amino-2-oxo-2,3-dihydropyrimidine bis(bis(2-thioxo-1,3-dithiole-4,5-dithiolato)-nickel)	4-aminopyrimidin-2(1H)-one bis(2-thioxo-1,3-dithiole-4,5-dithiolato)-nickel $\langle 1^+:1/1:1^-\rangle$	$CytH^+Cyt[NiL_2][NiL_2]^-$	UDIKUP
6-Amino-2-oxo-2,3-dihydropyrimidin-1-ium 6-amino-2-oxo-2,3-dihydropyrimidine bis(bis(2-thioxo-1,3-dithiole-4,5-dithiolato)-nickel)	4-aminopyrimidin-2(1H)-one bis(2-thioxo-1,3-dithiole-4,5-dithiolato)-nickel $\langle 1^+:1/1:1^-\rangle$	$CytH^+Cyt[NiL_2][NiL_2]^-$	UDIKUP01
6-Amino-2-oxo-2,3-dihydropyrimidin-1-ium 6-amino-2-oxo-2,3-dihydropyrimidine bis(2-thioxo-1,3-dithiole-4,5-dithiolato)-nickel	4-aminopyrimidin-2(1H)-one bis(2-thioxo-1,3-dithiole-4,5-dithiolato)-nickelate $\langle 1^+:1/1^-\rangle$	$CytH^+Cyt[NiL_2]^-$	UDILIE
Cytosinium cytosine hexafluorophosphate	cytosine hexafluorophosphate $\langle 1^+:1/1^-\rangle$	$CytH^+Cyt[PF_6]^-$	YUMGOD
bis(Hemicytosinium) bromide monohydrate	cytosine bromide water $\langle 1^+:1/1^-/1\rangle$	$CytH^+CytBr^-\cdot H_2O$	ZEYJOE
bis(Hemicytosinium) chloride monohydrate	cytosine chloride water $\langle 1^+:1/1^-/1\rangle$	$CytH^+CytCl^-\cdot H_2O$	ZEYJUK
catena-(pentakis(μ_2-aquo)-triaqua-bis(cytosine)-(μ_2-decavanadate)-trisodium tris(6-amino-2-oxo-2,3-dihydropyrimidin-1-ium) cytosine dihydrate)	sodium 4-aminopyrimidin-2(1H)-one decavanadate water $\langle 3^+/3^+:3/1^{6-}/10\rangle$	$(Na^+)_3(CytH^+)_3Cyt_3(V_{10}O_{28})^{6-}\cdot 10H_2O$	BUPMAB
Cytosinium hemi(tetrachloro-zinc(ii)) cytosine	cytosine tetrachlorozincate(II) $\langle 2^+:2/1^{2-}\rangle$	$(CytH^+)_2Cyt_2[ZnCl_4]^{2-}$	CYTZNC
trans-Tetra-aqua-bis(cytosine-O)-manganese(ii) diperchlorate cytosine solvate dihydrate	trans-tetraaqua-bis(cytosine-O)-manganese(II) cytosine perchlorate water $\langle 1^{2+}/2/2^-/2\rangle$	$[Mn(Cyt)_2(H_2O)_4]^{2+}Cyt_2(ClO_4^-)_2\cdot 2H_2O$	MAZGID
trans-Tetra-aqua-bis(cytosine-O)-cobalt(ii) diperchlorate cytosine solvate dihydrate	trans-tetraaqua-bis(cytosine-O)-cobalt(II) cytosine perchlorate water $\langle 1^{2+}/2/2^-/2\rangle$	$[Co(Cyt)_2(H_2O)_4]^{2+}Cyt_2(ClO_4^-)_2\cdot 2H_2O$	MAZGOJ

Systematic or common names were retained accordingly to the reported names. Note the middle dot character for proper radical description(LIWSIU, ODICOU).
